# Trends in the Use and Complications of Cardiac Resynchronization Therapy Device Implantation in Chronic Kidney Disease Patients

**DOI:** 10.19102/icrm.2023.14023

**Published:** 2023-02-15

**Authors:** Asim Kichloo, Dhanshree Solanki, Ronald Berger, Shakeel Jamal, Michael Albosta, Michael Aljadah, Muhammad Zia Khan, Khalil Kanjwal

**Affiliations:** ^1^Department of Internal Medicine, Central Michigan University, Saginaw, MI, USA; ^2^Department of Internal Medicine, Samaritan Medical Center, Watertown, NY, USA; ^3^Department of Health Administration, Rutgers University, New Brunswick, NJ, USA; ^4^Division of Cardiology, The Johns Hopkins University School of Medicine, Baltimore, MD, USA; ^5^Department of Internal Medicine, University of Miami, Jackson Memorial Hospital, Miami, FL, USA; ^6^Department of Internal Medicine, Medical College of Wisconsin, Milwaukee, WI, USA; ^7^Department of Internal Medicine, West Virginia University, Morgantown, WV, USA; ^8^Section of Electrophysiology, Michigan State University, McLaren Greater Lansing Hospital, Lansing, MI, USA

**Keywords:** Cardiac resynchronization therapy, chronic kidney disease, defibrillator, pacemaker

## Abstract

Large-scale multi-hospital data on cardiac resynchronization therapy (CRT) device implantation in patients with chronic kidney disease (CKD) are currently lacking. The purpose of this study was to examine the incidence of CRT device implantation in patients hospitalized with CKD and the impact of CRT device implantation on hospital complications and outcomes. We analyzed the Nationwide Inpatient Sample from 2008–2014 to identify yearly trends in CRT device implantation during CKD hospitalizations. We compared CRT biventricular pacemakers (CRT-Ps) and CRT defibrillators (CRT-Ds). We also obtained rates of comorbidities and complications associated with CRT device implantations. From 2008–2014, the proportion of hospitalized patients with a concurrent diagnosis of CKD receiving CRT-P devices consistently went up from 2008 to 2014 (from 12.3% to 23.8%, *P* < .0001) compared to the number of hospitalized patients with a concurrent diagnosis of CKD receiving CRT-D devices, which showed a consistent downward trend (from 87.7% to 76.2%, *P* < .0001). During CKD hospitalizations, most CRT device implantations were performed in patients aged 65–84 years (68.6%) and in men (74.3%). The most common complication of CRT device implantation during hospitalizations involving CKD was hemorrhage or hematoma (2.7%). Patients hospitalized with CKD who developed any complication associated with CRT device implantation had 3.35-fold increased odds of mortality compared to those without complications (odds ratio, 3.35; 95% confidence interval, 2.18–5.16; *P* < .0001). In summary, this study shows that CRT-P implantations became more common in CKD patients, while the rate of CRT-D implantations decreased over time. Hemorrhage or hematoma was the most common complication (2.7%), and the mortality risk was increased by 3.35 times in patients who developed periprocedural complications.

## Introduction

Chronic kidney disease (CKD) is defined by a glomerular filtration rate (GFR) of <60 mL/min/1.73 m^2^ for >3 months and having ≥1 markers of kidney damage, including albuminuria, urinary sediment abnormalities, tubular dysfunction, histologic abnormalities, structural abnormalities detected on imaging, or a history of kidney transplantation.^1^ It is estimated to affect >47 million people in the United States.^2^ In addition to secondary endocrine and metabolic dysfunction, patients with CKD have a significantly elevated risk of cardiovascular diseases, including heart failure, stroke, coronary artery disease, and atrial fibrillation.^1–3^

Cardiac resynchronization therapy (CRT) is indicated in patients with an ejection fraction of <35%, patients with left bundle branch block and a QRS duration of ≥ 150 ms, and patients with New York Heart Association class ≥II symptoms despite being on guideline-directed medical therapy for ≥3 months.^4^ Because of the elevated risk of heart disease, especially heart failure, in patients with CKD, these devices are presumed to be beneficial for prolonging life and reducing mortality. However, there is a paucity of large-scale, multi-hospital data regarding the current use and effects of CRT device implantation in patients hospitalized for CKD. The purpose of this study was to examine the incidence of CRT device implantation in patients hospitalized with CKD as well as the impact of CRT device implantation on hospital complications and outcomes.

## Methods

Data were obtained from the Nationwide Inpatient Sample (NIS) database published by the Healthcare Cost and Utilization Project (HCUP). The NIS database is the largest publicly available all-payer inpatient health care database in the United States and contains data from >7 million hospital stays each year.^5,6^ Stratified random sampling ensures that the NIS is representative of the U.S. population. National estimates can be obtained using the discharge weights assigned to the hospitalization records.^7^ Institutional review board approval was not needed for this study, as all patient information is de-identified within the NIS. A detailed overview of HCUP NIS is available at https://www.hcup-us.ahrq.gov/nisoverview.jsp.

**Figure S1:**
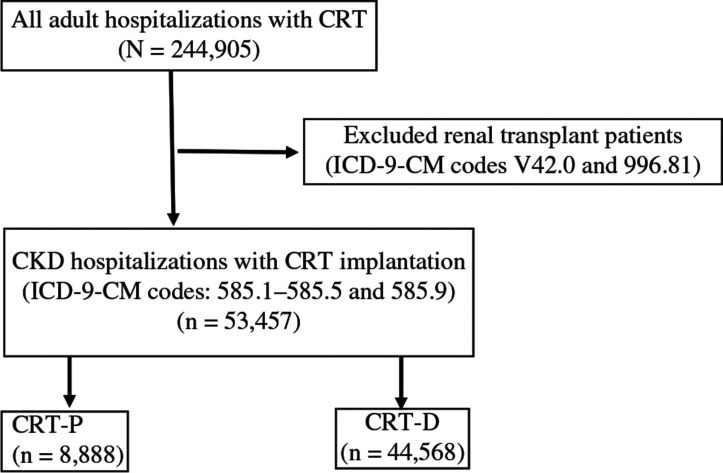
Graphical representation of the study design. *Abbreviations:* CKD, chronic kidney disease; CRT, cardiac resynchronization therapy; CRT-D, cardiac resynchronization therapy device with defibrillator; CRT-P, cardiac resynchronization therapy device with pacemaker; ICD-9-CM, International Classification of Diseases, Ninth Revision, Clinical Modification.

We excluded hospitalizations of patients <18 years of age. We used International Classification of Diseases, Ninth Revision, Clinical Modification (ICD-9-CM) codes to identify patients with a primary procedure code for CRT-P (biventricular pacemaker only, code 00.50) or CRT-D (biventricular pacemaker with a defibrillator, code 00.51) implantation. These codes have been used in previous NIS-based studies.^8,9^ We excluded patients with renal transplant (ICD-9-CM codes V42.0 and 996.81) from the cohort of hospitalizations for CRT implantation. We then identified patients hospitalized with a concurrent CKD diagnosis using ICD-9-CM diagnosis codes 585.1–585.5 (CKD 1–5, respectively) and 585.9 (CKD, unspecified). **Figure S1** shows a graphical representation of the sequentially derived study cohort. This method has been used in other NIS-based studies to correctly identify patients hospitalized with CKD.^10,11^

All hospitalizations were stratified according to age, sex, race, elective versus non-elective admission, disposition status, comorbidities, and complications. The description of these variables can be found in **Table S1**. All analyses were performed using SAS version 9.3 (SAS Institute Inc., Cary, NC, USA). Relevant hospital- and discharge-level weights were applied to the dataset to estimate the total number of hospitalizations involving CRT-P and CRT-D device implantations during hospitalizations involving a concurrent diagnosis of CKD in the United States from 2008–2014. Outcomes were compared using the chi-squared test for categorical variables and Student’s *t* test or linear regression analysis for continuous variables.

## Results

**Figure 1:**
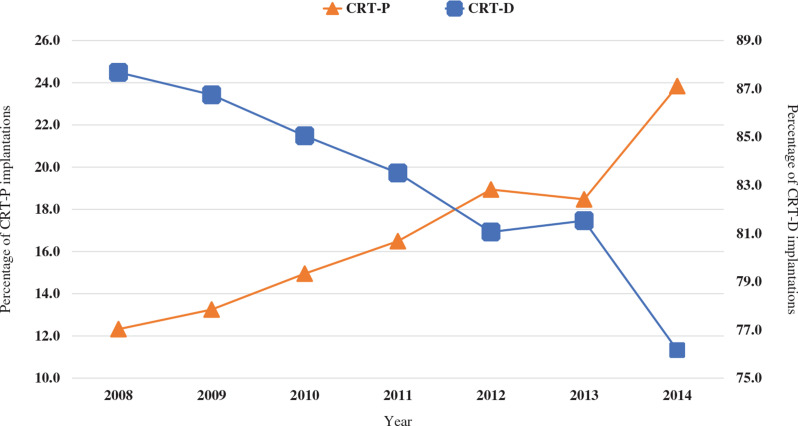
Trends in cardiac resynchronization therapy device implantations. *Abbreviations:* CRT-D, cardiac resynchronization therapy device with defibrillator; CRT-P, cardiac resynchronization therapy device with pacemaker.

Baseline patient-level and hospital-level characteristics, along with comorbidities in CKD patients who underwent CRT-D or CRT-P device implantation, are listed in **Tables 1 and 2**. From 2008–2013, there were a total of 53,457 CRT devices placed in patients with a concomitant diagnosis of CKD, among which 8,888 were CRT-P devices (16.6%) and 44,568 were CRT-D devices (83.4%) **(Table 1)**. During the study period, the number of patients receiving CRT devices who were hospitalized with a concurrent diagnosis of CKD almost doubled (from 15.8% in 2008 to 31.0% in 2014, *P* < .0001). Over time, the percentage of CKD patients receiving CRT-P devices increased (from 12.3% to 23.8%, *P* < .0001), whereas that of patients receiving CRT-D devices decreased (from 87.7% to 76.2%, *P* < .0001) **(Figure 1)**. The mean ages for CRT-P and CRT-D device implantation were 79.3 ± 0.2 years and 72.1 ± 0.1 years, respectively. Most implantations occurred in patients aged 65–84 years (68.6%) and in men (74.3%).

The most common comorbidities, aside from congestive heart failure (79.7%), were hypertension (79.2%), coronary artery disease (75.2%), diabetes mellitus (48.0%), chronic obstructive pulmonary disease (22.2%), and morbid obesity (5.23%). The overall mortality rate was 1.2%. The mortality rate was higher after CRT-P device implantation than after CRT-D device implantation (1.4% vs. 1.1%, *P* < .0001). The mean hospital length of stay (LOS) was longer (7.6 ± 0.2 days) for hospitalizations that included CRT-D device implantations compared to CRT-P device implantations (7.0 ± 0.3 days) (*P* < .0001). Most CRT recipients were discharged home (83.6%) **(Table 2)**.

**Figure 2:**
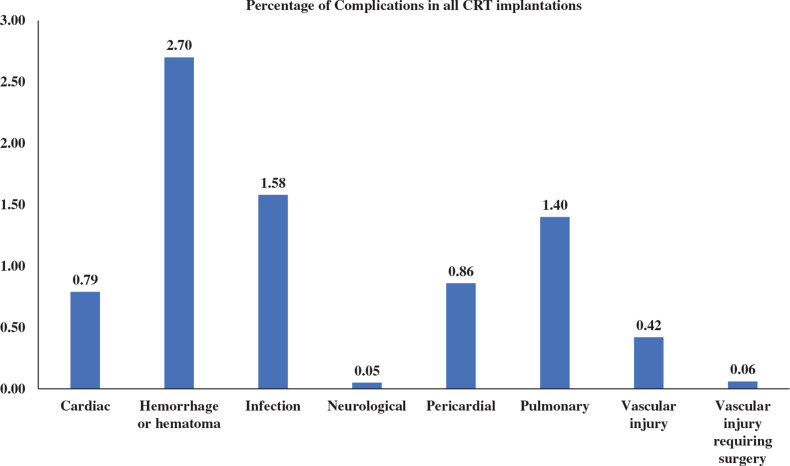
Percentage of complications in all cardiac resynchronization therapy device implantations.

The most common complication during CRT device implantation in patients hospitalized with a concurrent diagnosis of CKD was hemorrhage or hematoma (2.7%), while neurological complications (0.05%) were least frequently observed **(Figure 2 and Table 3)**. In addition to hemorrhage and hematoma, CRT device recipients in both study arms were found to have higher rates of infection, pericardial complications, and pulmonary complications compared to other complications. After controlling for confounding variables, patients hospitalized with a diagnosis of CKD who developed any complication associated with CRT device implantation had 3.35-fold increased odds of mortality compared to those without complications (odds ratio, 3.35; 95% confidence interval, 2.18–5.16; *P* < .0001) **(Table 4)**. Age, sex, and hospital characteristics had no association with inpatient mortality.

## Discussion

The main findings attained in this study after the incidence and outcomes of patients hospitalized with a diagnosis of CKD who underwent CRT device placement were evaluated are as follows. First, the incidence of CRT-D implantation was much greater than that of CRT-P implantation; however, CRT-P implantations increased over time, while the use of CRT-D devices consistently down-trended during the study period. Second, patients undergoing CRT-P implantation had higher rates of inpatient mortality; however, the LOS was greater for patients receiving CRT-D devices. Third, the most common complication of CRT implantation was hemorrhage/hematoma, followed by infection, pulmonary complications, and pericardial complications. Finally, patients who developed any complication had 3.35-fold greater odds for mortality compared to those without complications.

In this large, national database study, we found that the majority of CRT devices being implanted in patients with CKD were CRT-D devices. While this is consistent with data from previous studies evaluating the implantation trends of CRT devices with and without defibrillators,^12^ there may be a physiologic explanation for the increased need for CRT-D in patients with CKD. It is well known that patients with CKD have an increased preponderance for sudden cardiac death via life-threatening arrhythmias.^13^ The predisposition for these arrhythmias stems from elevated rates of coronary artery disease secondary to risk factors such as hypertension and diabetes; however, patients also have higher rates of heart failure and left ventricular hypertrophy as a result of long-standing hemodynamic overload due to elevated blood pressure, anemia, electrolyte disturbances, and uremia.^13,14^ In addition, a 2019 study found that CRT-D is superior to CRT-P with regard to reducing overall mortality, total mortality in heart failure hospitalizations, and total mortality related to major adverse cardiovascular events in patients with a GFR of <60 mL/min/1.73 m^2^.^15^ Because of this, it is reasonable to expect that the incidence of CRT-D implantation, especially in patients with CKD, would be higher. The reasoning behind the steady increase in the number of CRT-P devices being implanted during the study period is not readily apparent. Guidelines published in 2008 from the American College of Cardiology, American Heart Association, and Heart Rhythm Society did not favor the use of CRT-P over CRT-D or vice versa.^16^ Furthermore, clinical trials directly comparing both device modalities are lacking, although studies such as the Comparison of Medical Therapy, Pacing, and Defibrillation in Heart Failure (COMPANION) trial have reported some increased mortality benefit with CRT-D implantation over CRT-P implantation when comparing both to goal-directed medical therapy.^17^ One consideration is that the second Multicenter Automated Defibrillator Implantation Trial (MADIT-II) substudy found that patients with severe renal dysfunction (blood urea nitrogen ≥ 50 mg/dL and/or creatinine ≥ 2.5 mg/dL) did not demonstrate a benefit from ICD implantation.^18^ Additionally, the Re-evaluation of Optimal Re-synchronization Therapy in Patients with Chronic Heart Failure (RESET-CRT) and Danish Study to Assess the Efficacy of ICDs in Patients with Non-ischemic Systolic Heart Failure on Mortality (DANISH) trials showed no benefit of ICD in patients with non-ischemic cardiomyopathy. Because guidelines do not suggest a benefit with one treatment modality over the other, in addition to the increased costs and potential side-effects of the defibrillator portion of the CRT device, it may be that physicians and patients are choosing CRT-P devices more frequently in a shared decision-making model.

We found that patients receiving CRT-P devices had a higher rate of inpatient mortality when compared to CKD patients receiving CRT-D devices (1.4% vs. 1.1%). There are several possible reasons for this finding. First, patients receiving CRT-P devices were more likely to be older, with an average age of 79.3 ± 0.2 years compared to 72.1 ± 0.1 years found in patients receiving CRT-D devices. We also found that 33.9% of patients receiving CRT-P devices were >85 years of age compared to 8.5% of patients receiving CRT-D devices. It is reasonable to assume that hospitalized patients of significantly more advanced ages would experience worse inpatient mortality rates compared to their younger counterparts. In addition, our CRT-P device recipients likely have lower life expectancies due to their older age and comorbidity burden, and they may not qualify for implantation of a defibrillator based on their 1-year expected survival.^19^ Finally, patients with CRT-P implantation were not only more likely to suffer from mortality but also more likely to require discharge to a facility for further care after their inpatient stay (23.1% vs. 13.8%). This again signifies that the patients who received CRT-P devices had higher morbidity and mortality rates.

In our study, we found that the most common complication in patients receiving CRT devices was hemorrhage/hematoma. This is consistent with prior data demonstrating that CKD patients with albuminuria have an increased risk of hemorrhage, with the risk increasing as the GFR decreases.^20^ The pathophysiology behind the increased bleeding risk in patients with CKD is complicated and likely related to multiple mechanisms, including platelet dysfunction, impaired platelet–vessel wall interaction, anemia, and reduced clearance of certain medications that may prolong bleeding time.^21^ In addition, patients with cardiac diseases, such as coronary artery disease, atrial fibrillation, and heart failure, are more likely to be taking regular anticoagulation/anti-platelet therapies that may also contribute to the increased risk of bleeding.

Following hemorrhage/hematoma, the next most common complications included infection, pulmonary complications, and pericardial complications (see **Table 3** for a description of the complications). Infection during cardiac device implantation is a serious complication associated with significant morbidity and mortality rates. Prior to the study period, the incidence of infection during cardiac device implantation increased between the years 1993 and 2008 (from 1.5% to 2.41%).^22^ A recent systematic review and meta-analysis attempting to identify risk factors for implantable cardiac device infection found that several risk factors, including renal disease, heart failure, and anticoagulant use (among others), were associated with significant rates of infection.^23^ This is an important consideration in the context of the current study, as we have identified that our patient population is likely to have all of these risk factors on presentation. Therefore, minimization of any additional risk factors that may contribute to device-related infection, such as a lack of antibiotic prophylaxis, the need for replacement or revision procedures, increased procedure duration, and the need for temporary pacing, should be avoided in hospitalized patients if possible.^23^

Pulmonary complications, especially pneumothorax or hemothorax, are well-documented complications that may occur as a result of cardiac device implantation. In a multicenter study evaluating sex differences in complication rates secondary to CRT implantation, it was found that women were significantly more likely than men to suffer from pneumothorax and hemothorax.^19^ This finding was attributed to differences in body composition, including lower body mass index values and smaller vascular and cardiac dimensions, in female patients Providers should take caution when implanting these devices in order to minimize the occurrence of these complications.

Pericardial complications, such as hemopericardium, cardiac tamponade, and pericarditis, are additional well-known device-related complications. A previous study using NIS data evaluated risk factors for the development of cardiac tamponade in patients undergoing CRT implantation and found that those with coagulation disorders, which included the use of long-term anticoagulation and antiplatelet therapies, were more likely to develop cardiac tamponade.^24^ We previously reported that patients with CKD have higher rates of coronary artery disease, heart failure, and arrhythmias, which may require regular use of anticoagulants and antiplatelet therapies. Interestingly, the aforementioned study did not find a statistically significant increase in the risk for tamponade in patients with documented renal disease.^24^ In addition, implantation of the right ventricular lead at the apex has been associated with a higher risk of cardiac perforation and tamponade compared to septal placement.^24^ Physicians need to be careful when performing CRT lead placement to avoid vessel dissection or perforation and should consider the placement of septal leads when possible to avoid damage to the ventricular wall that could result in perforation.

Finally, patients who developed any implant-related complication had a >3-fold increased risk of in-hospital mortality. This finding is not necessarily surprising, as the development of complications secondary to procedural intervention in hospitalized patients would expectedly increase morbidity and mortality. However, this is an important finding as physicians should pay careful attention to patients who are at an increased risk for developing complications and weigh the risk–benefit ratio of CRT implantation in the inpatient setting. Most inpatient CRT implantations are non-emergent; thus, it may be preferable that physicians delay the procedure to a time when patients are more clinically stable and have recovered from any acute decompensation that led to their initial hospitalization.

In this study, we performed a retrospective analysis by collecting data from an administrative database using ICD discharge codes. A strength of our study is its large sample size (N = 53,457) of CRT device implantations in patients hospitalized with CKD. Because of this and the time period over which we studied patients, we were able to appreciate trends in outcomes and complications in our study population. However, there are limitations to our study. The usage of the NIS database relies on proper documentation of ICD-9 codes by providers. Therefore, our study is subject to missing data on account of inappropriate provider documentation. To account for this, we have used ICD-9 codes that have been validated in prior studies. In addition, because these procedures were performed in hospitalized patients, the results are not generalizable to patients with CKD electing to undergo CRT implantation in the outpatient setting. Lastly, our study reports trends regarding outcomes and complications that occurred during the inpatient stay and would not reflect complications or outcomes occurring after discharge.

## Conclusion

Hospitalized patients with a concurrent CKD diagnosis who underwent CRT implantation were more likely to receive CRT-D devices than CRT-P devices, although the yearly trends showed an increase in CRT-P implantations and a decrease in CRT-D implantations during the study period. Patients who underwent CRT-P device implantation had a higher inpatient mortality rate, likely secondary to having higher-risk features at baseline. Hemorrhage/hematoma was a common device-related complication, and patients developing any device-related complication had a 3-fold increase in mortality.

## Figures and Tables

**Table 1:** Trends in the Implantation of Cardiac Resynchronization Therapy Devices with Pacemakers Versus Defibrillators in Chronic Kidney Disease Hospitalizations

	2008	2009	2010	2011	2012	2013	2014	Total
**Overall CRT implantations in the United States by year**	44,876	47,293	39,311	35,915	31,110	25,855	20,545	244,905
**All CRT implantations in patients with CKD**								
n (% of yearly total)	7,076 (15.8)	8,946 (18.9)	8,185 (20.8)	8,720 (24.3)	7,575 (24.3)	6,580 (25.4)	6,375 (31.0)	53,457 (21.8)
**CRT-P**								
n (% of yearly total)	872 (12.3)	1,185 (13.3)	1,223 (15.0)	1,438 (16.5)	1,435 (18.9)	1,215 (18.5)	1,520 (23.8)	8,888 (16.6)
**CRT-D**								
n (% of yearly total)	6,204 (87.7)	7,761 (86.8)	6,961 (85.1)	7,282 (83.5)	6,140 (81.1)	5,365 (81.5)	4,855 (76.2)	44,568 (83.4)

*Abbreviations:* CKD, chronic kidney disease; CRT, cardiac resynchronization therapy; CRT-D, cardiac resynchronization therapy device with defibrillator; CRT-P, cardiac resynchronization therapy device with pacemaker.

**Table 2:** Baseline Characteristics: Cardiac Resynchronization Therapy Implantations in Chronic Kidney Disease Hospitalizations

	CRT-P	CRT-D	All CRT	*P* Value
Weighted number (n)	8,888	44,568	53,457	
Mean age ± SE, years	79.3 ± 0.2	72.1 ± 0.1	73.3 ± 0.1	
Age, years (%)				<.0001
18–44	0.3	1.5	1.3	
45–64	6.1	19.6	17.3	
65–84	59.7	70.4	68.6	
≥85	33.9	8.5	12.7	
Sex (%)				<.0001
Male	60.9	77.0	74.3	
Female	39.1	23.0	25.7	
Race (%)				<.0001
White	79.7	73.4	74.4	
Black	10.4	15.7	14.9	
Hispanic	5.2	6.2	6.1	
Others	4.7	4.7	4.7	
Type of admission (%)				.0004
Emergent or urgent	96.5	95.7	95.9	
Elective	3.5	4.3	4.2	
Disposition status (%)				<.0001
Home	75.5	85.2	83.6	
Facility	23.1	13.8	15.3	
Died	1.4	1.1	1.2	
Mean LOS	7.0 ± 0.3	7.6 ± 0.2	6.9 ± 0.1	
Comorbidities (%)				
COPD	23.5	21.9	22.2	.0006
Congestive heart failure	77.4	80.2	79.7	<.0001
Coronary artery disease	66.79	76.9	75.21	<.0001
Diabetes mellitus	43.8	48.8	48.0	<.0001
Hypertension	80.4	78.94	79.18	.001
Morbid obesity	4.76	5.33	5.23	.01

*Abbreviations:* CKD, chronic kidney disease; COPD, chronic obstructive pulmonary disease; CRT, cardiac resynchronization therapy; CRT-D, cardiac resynchronization therapy device with defibrillator; CRT-P, cardiac resynchronization therapy device with pacemaker; LOS, length of stay; SE, standard error.

**Table 3:** Rates of Complications: Cardiac Resynchronization Therapy Devices with Pacemakers Versus Defibrillators Versus All Cardiac Resynchronization Therapy Devices in CKD Hospitalizations

	CRT-P	CRT-D	All CRT	*P* Value
Cardiac	0.39	0.87	0.79	<.0001
Hemorrhage or hematoma	2.62	2.72	2.70	.29
Infection	1.06	1.69	1.58	<.0001
Neurological	0.06	0.05	0.05	.83
Pericardial	1.26	0.78	0.86	<.0001
Pulmonary	1.64	1.35	1.40	.38
Vascular injury	0.34	0.44	0.42	.23
Vascular injury requiring surgery	0.11	0.06	0.06	.13

*Abbreviations:* CRT, cardiac resynchronization therapy; CRT-D, cardiac resynchronization therapy device with defibrillator; CRT-P, cardiac resynchronization therapy device with pacemaker.

**Table 4:** Predictors of Mortality in Chronic Kidney Disease Hospitalizations with Cardiac Resynchronization Therapy Implantations

Variables	OR (95% CI)	*P* Value
**Age, years**		
18–64	[Reference]	
≥65	0.99 (0.62–1.56)	.95
**Sex**		
Male	1.11 (0.73–1.67)	.64
Female	[Reference]	
**Hospital bed size**		
Small	[Reference]	
Medium	1.48 (0.60–3.64)	.39
Large	2.01 (0.89–4.54)	.09
**Hospital region**		
Northeast	[Reference]	
Midwest	1.15 (0.69–1.91)	.59
South	0.95 (0.58–1.54)	.82
West	0.69 (0.36–1.30)	.25
**Hospital type**		
Rural	[Reference]	
Urban non-teaching	0.81 (0.39–1.69)	.58
Teaching	0.60 (0.30–1.22)	.16
**Complications**		
No complication	[Reference]	
Any complication	3.35 (2.18–5.16)	< .0001

*Abbreviations:* CI, confidence interval; CKD, chronic kidney disease; CRT, cardiac resynchronization therapy; OR, odds ratio. ^a^C-statistic = 0.63.

**Table S1:** ICD-9-CM Codes, CCS Codes, and Procedural Codes

Procedure	Procedural Code
CRT-P	00.50
CRT-D	00.51

*Abbreviations:* CRT-D, cardiac resynchronization therapy device with defibrillator; CRT-P, cardiac resynchronization therapy device with pacemaker.

**Table:** 

Disease	Type of Code	Code(s)
CKD	ICD-9-CM	585.1–585.5, 585.9
Morbid obesity	ICD-9-CM	278.01
Renal transplant recipient	ICD-9-CM	V42.0, 996.81
CHF	CCS	108
COPD	CCS	127
Coronary artery disease	CCS	101
Diabetes mellitus	CCS	49, 50
Hypertension	CCS	98, 99

Abbreviations: CCS, Clinical Classification Software; CHF, congestive heart failure; CKD, chronic kidney disease; COPD, chronic obstructive pulmonary disease; ICD-9-CM, International Classification of Diseases, Ninth Revision, Clinical Modification.

**Table:** 

Complications	ICD-9-CM Code(s)
**Pericardial**	
Hemopericardium	423
Tamponade	423.3
Pericardiocentesis	37
Acute pericarditis	420.9
Unspecified disease of pericardium	423.9
**Cardiac**	
Arrest during or resulting from a procedure	997.1
Insufficiency during or resulting from a procedure	997.1
Cardiorespiratory failure during or resulting from a procedure	997.1
Heart failure during or resulting from a procedure	997.1
**Pulmonary**	
Pneumothorax	512.1
Hemothorax	511.8
Chest tube	34.04
Other iatrogenic pulmonary complications	997.39
**Hemorrhage/hematoma**	
Hemorrhage/hematoma complicating a procedure	998.11
Acute posthemorrhagic anemia	285.1
**Vascular**	
Accidental puncture or laceration during a procedure	998.2
Vascular complication requiring surgical repair	39.31, 39.41, 39.49, 39.52, 39.53, 39.56, 39.57, 39.58, 39.59, 39.79
**Infection**	
Infection and inflammatory reaction due to cardiac device, implant, and graft	996.61
Postprocedural aspiration pneumonia	997.32
**Neurological**	
Nervous system complication, unspecified	997
Central nervous system complication	997.01
Iatrogenic cerebrovascular infarction or hemorrhage	997.02
Other nervous system complications	997.09
Transient ischemic attack	435.9

*Abbreviation:* ICD-9-CM, International Classification of Diseases, Ninth Revision, Clinical Modification.
