# Erratum to Kichloo A, Solanki D, Berger R, et al. Trends in the Use and Complications of Cardiac Resynchronization Therapy Device Implantation in Chronic Kidney Disease. *J Innov Cardiac Rhythm Manage*. 2023;14(2):5339–5347

**DOI:** 10.19102/icrm.2023.140310

**Published:** 2023-03-15

In the article titled “Trends in the Use and Complications of Cardiac Resynchronization Therapy Device Implantation in Chronic Kidney Disease Patients” by Kichloo et al. published in the February 2023 issue of *The Journal of Innovations in Cardiac Rhythm Management*, a note about Figure S2 inadvertently was left in the text before the references. No such figure exists.

Additionally, reference 20 was inadvertently left in the text; it should not exist, and all citation and associated reference numbers thereafter should be reduced by 1.

Finally, in the following sentence on page 5345 in the Discussion section, the reference to Table S1 should instead be a reference to Table 3.

*“Following hemorrhage/hematoma, the next most common complications included infection, pulmonarycomplications, and pericardial complications (see **Table S1** for a description of the complications).”*

The online record of this article has been corrected accordingly.

